# Gene 33/Mig6/ERRFI1, an Adapter Protein with Complex Functions in Cell Biology and Human Diseases

**DOI:** 10.3390/cells10071574

**Published:** 2021-06-22

**Authors:** Dazhong Xu, Cen Li

**Affiliations:** Department of Pathology, Microbiology and Immunology, New York Medical College School of Medicine, Valhalla, NY 10595, USA; cen_li@nymc.edu

**Keywords:** gene 33, ERRFI1, adapter protein, DNA damage, EGFR, cancer, signal transduction

## Abstract

Gene 33 (also named Mig6, RALT, and ERRFI1) is an adapter/scaffold protein with a calculated molecular weight of about 50 kD. It contains multiple domains known to mediate protein–protein interaction, suggesting that it has the potential to interact with many cellular partners and have multiple cellular functions. The research over the last two decades has confirmed that it indeed regulates multiple cell signaling pathways and is involved in many pathophysiological processes. Gene 33 has long been viewed as an exclusively cytosolic protein. However, recent evidence suggests that it also has nuclear and chromatin-associated functions. These new findings highlight a significantly broader functional spectrum of this protein. In this review, we will discuss the function and regulation of Gene 33, as well as its association with human pathophysiological conditions in light of the recent research progress on this protein.

## 1. Introduction

**Figure 1:**
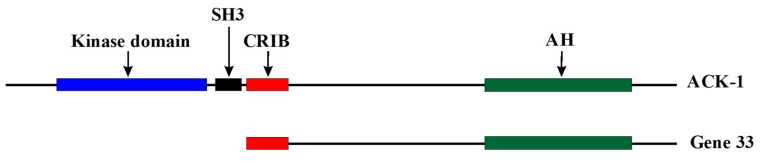
Comparison of linear structures of Gene 33 and ACK-1. SH3: Src homology 3 domain, CRIB: Cdc42/Rac interactive and binding domain. AH: Ack homology domain.

Gene 33 (also named Mig6, RALT, and ERRFI1) was discovered as a glucocorticoid-induced transcript from the rat liver using the differential hybridization technique in 1985 [1]. The gene that encodes this transcript was initially named *p33* and was later renamed *gene 33* to avoid confusion with its protein product [2]. The human homologue of *gene 33* was later identified from quiescent fibroblasts treated with serum and named as mitogen-inducible gene 6 (*Mig6* or *Mig-6*), for its high inducibility by serum [3]. Subsequent studies revealed that the transcript of *gene 33* can be induced by a wide variety of extracellular stimuli and is widely expressed [2,3,4,5,6,7,8,9,10,11,12]. The induction of *gene 33* by multiple signaling inputs is consistent with the fact that the promotor region of *gene 33* contains an array of regulatory elements [2,13]. *gene 33* is considered an immediate early response gene, defined as quick induction in response to stimuli without the requirement of de novo protein synthesis [3,14]. Gene 33 appeared rather late in the evolution, existing only in vertebrates. It shares considerable homology to the C-terminal portion of activated CDC42-associated kinase 1 (ACK1). Structurally, it is more or less an ACK1 without the kinase domain and the SRC-homology 3 domain (SH3) at the *N*-terminus (Figure 1). It is likely that Gene 33 was descended from ACK1 during evolution to fulfill the functional needs of more advanced animals.

Although the highly inducible nature of Gene 33 by multiple stimuli, particularly insulin, drew considerable interest in this gene soon after its discovery, the function of its protein product remained elusive until early 2000, when three studies were published on the function of this protein [4,15,16]. The laboratory of John Kyriakis showed that Gene 33 can be induced in the kidney after unilateral nephrectomy and/or streptozotocin-induced diabetes [4]. Gene 33 can also be induced by a mechanical strain in a JNK-dependent fashion in rat renal mesangial cells [4]. The study also described the domain structure of the protein and the potential signaling pathway connecting Gene 33, the small GTPase Cdc42, and JNK [4]. The laboratories of Oreste Segotto and Axel Ullrich independently discovered the interaction between rat Gene 33 with ErbB2 and Mig6 with EGFR, respectively, using the yeast two-hybrid system [15,16]. The interactions were found to inhibit the signaling pathways and fibroblast transformation mediated by these receptors [15,16]. These studies also showed that Gene 33 can also be induced by the activation of these receptors, establishing that Gene 33 is a potential feedback inhibitor of the signaling pathway mediated by these receptors. The Segotto group renamed Gene 33 as receptor-associated late transducer (RALT). The official name given later by the Human Genome Organization to this gene is ErbB receptor feedback inhibitor 1 (*ERRFI1*). This review will refer to this protein using its original common name “Gene 33” and the gene encoding it using its official name *ERRFI1* (*Errfi1* for the rodent gene).

The involvement of Gene 33 in the signaling of the ErbB family receptor tyrosine kinases (RTKs) sparked intense interest in this then little-known protein and led to a series of studies that solidified its role in the ErbB receptor signaling pathway [11,17,18,19,20,21,22,23,24,25,26]. Although the regulation of ErbB receptors appears to be the most prominent function of Gene 33, its involvement in other signaling pathways has also been revealed. The biological roles of Gene 33 in various pathophysiological processes have become clearer as well. Although Gene 33 has long been regarded as an exclusively cytoplasmic protein, recent evidence showed that a fraction of it is localized in the nucleus and associated with chromatin. This nuclear/chromatin fraction of Gene 33 has been shown to modulate the DNA damage response in response to genotoxic stresses [27,28]. These new findings significantly expand the functional profile of this protein. Several excellent and focused review articles on Gene 33 have been published in the past, with main emphases on its association with the ErbB family RTKs and its role in cancer [29,30,31,32]. This article intends to provide a more comprehensive view of this protein in light of the recent research progress.

## 2. Molecular Biology of Gene 33

**Figure 2:**
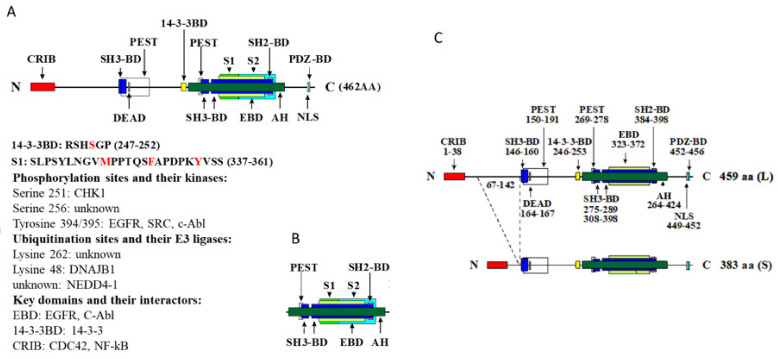
Linear structures of human and rat Gene 33. (**A**). Human Gene 33. Color-coded boxes represent different types of domains. CRIB: Cdc42/Rac interactive and binding domain, DEAD: DEAD box, PEST: PEST sequence, 14-3-3BD: 14-3-3 binding domain, SH3-BD: SH3 binding domain, EBD: EGFR or ErbB binding domain, SH2-BD: SH2 binding domain, AH: ACK homology domain, S1: segment 1, S2: segment 2, NLS: nuclear localization sequence, PDZ-BD: PDZ binding domain. The amino acid sequences of 14-3-3BD and segment 1 are shown with the key residues highlighted. Functionally significant phosphorylated residues and their kinases, key ubiquitination sites and their E3 ligase, as well as key domains and their known interactors are also listed. (**B**). Key features of the AH domain are shown. (**C**). Rat Gene 33 showing both the long and short splicing variants. The exact locations of domains are shown.

The linear structure of human Gene 33 represents a typical adapter/scaffold protein: containing multiple protein–protein interaction domains without having a discernable catalytic motif (Figure 2). These domains include a Cdc42/Rac-interactive binding (CRIB) domain, a 14-3-3-binding domain (14-3-3-BD), three proline-rich regions with potential to interact with the Src homology-3 domain (SH3-BD), a potential Src-homology-2-binding domain (SH2-BD), and a PDZ-binding domain (PDZ-BD). There are two stretches of PEST sequences, a putative DEAD Box and a putative nuclear localization signal (NLS). A region highly homologous to the non-receptor tyrosine kinase-activated CDC42 associated kinase 1 (AH) is located at the *C*-terminal portion of the protein, within which a minimum ErbB binding domain or region (EBD or EBR) is located (Figure 2A). 

The most functionally significant and well-characterized domain of Gene 33 characterized thus far is the EBD domain. The EBD domain is required for binding between Gene 33 and the ErbB (or EGFR) family receptor tyrosine kinases [11,15,16,18,22]. It also mediates the interaction between Gene 33 and the non-receptor tyrosine kinase c-Abl [27,33]. Two “segments” (namely segment 1 and 2 or AH1 and AH2) within the AH domain interact with the kinase domains of the EGFR and c-Abl in a fashion resembling the interaction between CDK and cyclin [22,33]. Segment 1 (AA337-361 in humans) is located within the *N*-terminal side of EBD whereas segment 2 (AA362-412 in humans) is C-terminal to segment 2 and extends outside EBD (Figure 2B) [22]. Three residues within segment 1 (M346, F352, and Y358 in humans) are critical for the interaction of Gene 33 with EGFR (Figure 2A) [22]. F352 and Y358 appear to also be required for the interaction of Gene 33 with c-Abl [27,33]. While both segments are required for high potency inhibition of EGFR, segment 2 is important for inhibition of the activated EGFR kinase domain [22]. It also plays a bigger role in the interaction between Gene 33 and c-Abl [33]. Interestingly, although EBD interacts with the kinase domains of EGFR and c-Abl in a very similar fashion, the functional consequences are very different. While the interaction with EGFR inhibits its kinase activity by preventing the formation of the activating dimer interface [22], the interaction with c-Abl increases its activity by disrupting the auto-inhibitory mechanism of c-Abl [33]. Gene 33 also mediates EGFR endocytosis and lysosomal degradation via the RALT endocytic domain (RED, AA 143-323) [21]. However, ectopic expression of Gene 33 has also been shown to prevent EGF-induced EGFR degradation [11,34]. This is likely due to inhibition of EGFR phosphorylation by excessive Gene 33, which limits phosphorylation-dependent degradation of EGFR [11]. The EBD domain has recently been shown to also promote the binding of Gene 33 to chromatin and for targeting c-Abl to chromatin [27].

The CRIB domain of Gene 33 has been shown to interact with CDC42, a Rho-like small GTPase, through which it activates the JNK pathway and inhibits HGF- and EGF-induced cell migration [4,35]. The CRIB domain may also interact with the NF-κB binding region of IκB, which shares strong sequence similarity to the CRIB motif [36]. This interaction prevents the inhibitory binding of IκB to the transcription factor NF-κB, thereby activating NF-κB [36]. The CRIB domain of Gene 33 has recently been shown to mediate the interaction of Gene 33 with AKT, particularly ATK2 and AKT3, and the AKT phosphatase PHLPP [37]. Binding of Gene 33 to AKT prevents the interaction between AKT and PHLPP, thereby avoiding dephosphorylation and inactivation of AKT by PHLPP [37]. In contrast, another report suggests that Gene 33 inhibits AKT phosphorylation through direct interaction in endometrial epithelial cells [38], suggesting a potential cell-type-dependent role of Gene 33 on AKT function.

The 14-3-3BD domain has been shown to bridge the binding of Gene 33 to the 14-3-3 protein [4,27,39]. This interaction facilitates the retention of Gene 33 in the cytosol [27]. The key serine residue within the 14-3-3BD (S251 in humans, Figure 2A) can be phosphorylated by the checkpoint kinase CHK1 upon EGF stimulation and the phosphorylation reduces the ability of Gene 33 to inhibit EGFR [39,40]. The PEST domains of Gene 33 are conceivably responsible for its degradation by the ubiquitin-proteasome system, consistent with the highly labile nature of Gene 33 [41]. 

Gene 33 has been shown to interact with the kinase domain of WEE1, an important regulator of mitosis, via its SH3-BD [42]. This interaction increases the activity of WEE1 by preventing the recruitment of the ubiquitin E3 ligase βTrCP-SCF and subsequent degradation by the proteasome [42]. The SH3-BD may also mediate the binding of Gene 33 with a number of important SH3 domain-containing signaling proteins, such as Grb2, Src, the p85 subunit of PI3K, PLCγ, and Fyn, with various affinities [15,24,29,35]. 

Gene 33 has been shown to interact with the adapter protein SHC1, thereby inhibiting its tyrosine phosphorylation at Tyr239/240 [43]. It may also interact with STAT3, thereby inhibiting its phosphorylation [44]. However, the domains of Gene 33 that mediate these interactions remain to be determined.

The function of the putative NLS remains elusive. Although Gene 33 has been found in the nucleus, the NLS motif does not seem to be important for its nuclear localization [27]. The functional significance of the DEAD box and the PDZ binding domain of Gene 33 are unknown. DEAD boxes are typically possessed by DEAD box proteins, which are RNA helicases that regulate RNA metabolism [45]. PDZ-BD should bind to the PDZ domain, which functions to facilitate the formation of multiprotein complex and anchoring of cell surface receptors to the actin cytoskeleton [46]. The existence of these sequences in Gene 33 suggests a more expanded functional spectrum of Gene 33. There is a confirmed splicing variant of rat Gene 33, which is missing a short stretch of amino acids in the *N*-terminal region [1] (Figure 2C). Although not extensively examined, the short form of Gene 33 presumably retains all the functions of the long form, as all the key domains are intact.

## 3. Regulation of Gene 33

*ERRFI1* is considered an immediate early response gene and its product is readily inducible by a wide array of extracellular and intracellular stimuli. This feature is supported by the presence of multiple regulatory elements at its promoter region, including the TPA response element (TRE), the cAMP response element (CRE), the glucocorticoid response element (GRE), the serum response element (SRE), and binding sites for specific protein 1 (SP-1) [2,13,23,47]. Consequently, the product of *ERRFI1* has been shown to be induced by factors, such as EGF, insulin, vasoactive peptides, glucocorticoids, progesterone, lysophosphatidic acid (LPA), osmotic shock, ER stress, hypoxia, and mechanical forces [2,4,7,8,11,14,17,23,48,49,50,51]. Intracellularly, Gene 33 expression is primarily mediated by MAPK pathways, particularly the MEK-ERK pathway, and the glucocorticoid receptor (GR) [11,17,23,41,52]. Other pathways, such as PKC and PI3K, may also participate in this process depending on the stimuli [17,41]. However, the involvement of the PI3K pathway in the induction of Gene 33 appears to be cell type dependent, as discussed in a previous review [29]. Of interest, Gene 33 can be induced by hypoxia, although no HIF-response element (HRE) presents in the Gene 33 promotor [5,17]. In neonatal rat cardiomyocytes, Gene 33 induction by hypoxia is dependent on both ERK and PI3K pathways, which closely resembles that triggered by platelet-derived growth factor (PDGF) [17]. 

Epigenetically, Gene 33 expression can be negatively regulated by a number of microRNAs, including miR-200, miR-148a, miR-126, miR-205, miR-214, miR-374, miR-589, and miR-2355 [28,53,54,55,56,57,58,59,60,61]. Promotor methylation of *ERRFI1* has been found in 79% of human papillary thyroid cancer patient specimens, which corresponds to reduced expression of Gene 33 [62]. However, no *ERRFI1* promoter methylation or histone acetylation were detected in selected lung cancer and melanoma cell lines [63]. Nonetheless, inhibition of the DNA methyltransferase or the histone deacetylase was able to alter the expression of Gene 33, albeit in a cell-type-dependent manner, suggesting that these mechanisms can indirectly affect the expression of Gene 33 [63]. On the other hand, inhibition of these enzymes had no effect on Gene 33 expression in breast cancer cells with low Gene 33 expression [24]. Thus, promoter modifications by means of DNA methylation and/or histone modifications affect Gene 33 expression in a cell-type-dependent manner. Missense and nonsense mutations as well as deletion of *ERRFI1* can also occur, particularly in cancers, to regulate the expression and/or functions of Gene 33. This aspect will be discussed later.

The labile nature of Gene 33 and the existence of PEST domains suggest that Gene 33 is subjected to regulation by the ubiquitin-proteasome system. This notion has been confirmed experimentally [28,41]. The lysine residue K362 was identified as a ubiquitination site of Gene 33 [64] (Figure 2A). DNAJB1, a small heat shock protein of the HSP40 family, was shown to promote K48 ubiquitination of Gene 33 [65]. Furthermore, the ubiquitin E3 ligase NEDD4-1 was reported to mediate ubiquitination and degradation of Gene 33 by the type I γ phosphatidylinositol phosphate 5-kinase i5 (PIPKIγi5) in the breast cancer cell MDA-MB-231 and the lung cancer cell H-1975 [66] (Figure 2A). Moreover, Gene 33 can be suppressed by hexavalent chromium (Cr(VI)) in part via the ubiquitin-proteasome system, although the E3 ligase that mediates the degradation remains to be determined [28]. Lastly, N-Myc downstream-regulated gene 1 (NDRG1) has been shown to post-transcriptionally stabilize Gene 33 [67].

**Figure 3:**
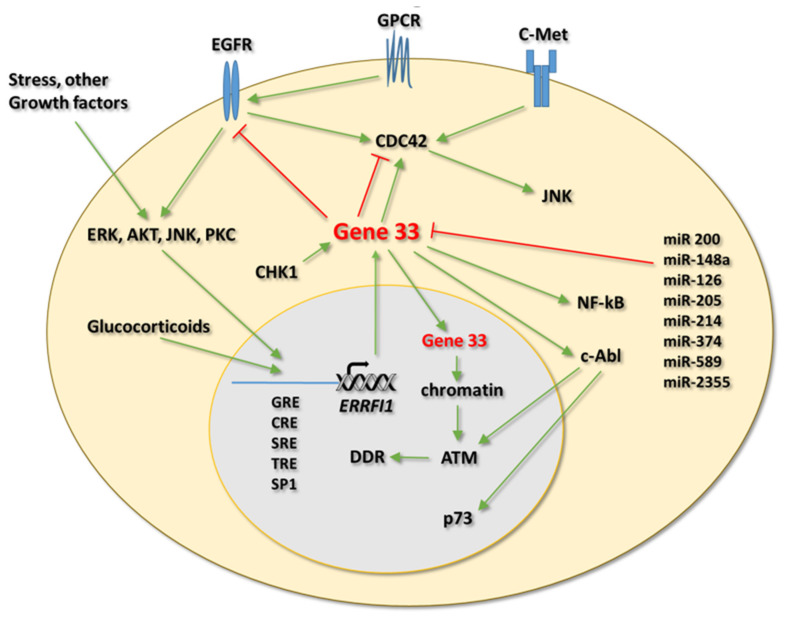
Main regulations and signaling of Gene 33. Abbreviations: DDR: DNA damage response, GPCR: G-protein-coupled receptor. Other abbreviations are Gene names. Green arrows represent activation, Red “T” bars represent inhibition.

Gene 33 is a highly phosphorylated protein, both at serine/threonine and tyrosine residues (PhosphoSitePlus, www.phosphosite.org, accessed on 4 August 2020). Phosphorylation of a number of serine/threonine and tyrosine residues in Gene 33 has been confirmed to be functionally relevant (Figure 2A). Serine 251, a key residue within the 14-3-3BD, can be phosphorylated by CHK1 [27,39,40]. Phosphorylation of this residue appears to help retain Gene 33 in the cytosol, likely by promoting its binding to 14-3-3 [27]. Phosphorylation of serine 256 is associated with active EGFR mutations (19del and L858R) and limits the ability of Gene 33 to promote ubiquitination and degradation of the wild-type EGFR [19]. Tyrosine phosphorylation of Gene 33 by EGFR at residues 394 and 395 was first reported to reduce its ability to inhibit the kinase activity of EGFR in vitro [68]. However, a more recent study showed that tyrosine phosphorylation of Gene 33, sequentially by Src and EGFR at residues 395 and 394, increased its binding and inhibition of both the wild-type and mutant (L858R) EGFR, thereby diminishing the transformation ability of this receptor [69]. Consistently, mutation of these two residues abolished the interaction between Gene 33 and mutant EGFR (19del and L858R) [34]. Of note, phosphorylation of tyrosine 395 by Src appears to prime the phosphorylation of tyrosine 394 by EGFR, as prior phosphorylation of tyrosine 395 greatly increases the ability of EGFR to phosphorylate tyrosine 394 [69]. The reason underlying the discrepancy between the two studies is unclear. Of note, tyrosine 394 and 395 are also targets of c-Abl. Phosphorylation of them by c-Abl facilitates the binding between Gene 33 and c-Abl [33]. Figure 3 illustrates the main regulations and intracellular signaling pathways of Gene 33. 

## 4. Biological Functions of Gene 33

### 4.1. Gene 33 in ErbB Signaling and Cell Proliferation

The finding that Gene 33 is inducible by EGFR activation and Gene 33 in turn inhibits EGFR qualifies it as a bona fide feedback inhibitor of the EGFR signal transduction pathway, adding it to a list of proteins with similar functions [30,31,70]. Given its role in direct inhibition of the ErbB family RTKs, it is conceivable that Gene 33 inhibits all the cellular activity downstream of these receptors. Thus, ectopic expression of Gene 33 inhibits G1 phase entry and transformation of murine fibroblasts induced by EGFR or ErbB2 [11,15,16]. Gene 33 has also been shown to mediate the feedback inhibition from mutated B-Raf to EGFR, thereby limiting transformation of murine fibroblasts induced by mutant B-Raf [71]. Gene 33 inhibits proliferation of breast cancer cells, chondral cells in the cartilage, endometrial cancer cells, thyroid cancer cells, and nucleus pulposus cell in the intervertebral disc [24,56,62,72,73,74,75,76]. Overexpression of Gene 33 inhibits proliferation of endometrial cancer cells [77].

Given that G-protein-coupled receptors often transactivate EGFR [70], it is not surprising that Gene 33 inhibits the signaling downstream of the endothelin receptor [11]. As Gene 33 can be induced by activation of GR, it is conceivable that Gene 33 contributes to the crosstalk between the glucocorticoid signaling and EGFR signaling. Thus, Gene 33 is induced by dexamethasone and in turn inhibits the EGFR signaling [11]. This mechanism may contribute to the long-standing observation that glucocorticoid signaling suppresses the EGFR pathway and cell proliferation [78]. This mechanism appears to be responsible for the inhibition of islet β cell proliferation by glucocorticoid [79]. Gene 33 has also been shown to inhibit estrogen-induced endometrial cell proliferation and mediates the suppressive effect of progesterone on the estrogen function [51].

An EGFR-independent effect of Gene 33 on cell proliferation has also been documented. A recent study demonstrated the involvement of Gene 33 in cell cycle progression by protecting the WEE1 kinase from proteasomal degradation [42]. As WEE1 is a key regulator of the G2/M checkpoint by phosphorylating and inhibiting CDK1 [80], Gene 33 may potentiate the checkpoint function of WEE1. Mechanistically, Gene 33 binds to WEE1, thereby preventing its interaction with the E3 ubiquitin ligase βTrCP [42]. This mechanism appears to be required for cell cycle arrest at the G2/M phase in response to DNA damage [42].

Although Gene 33 is generally considered an inhibitor of the mitotic signaling and cell proliferation, the effect is not always straightforward. In BEAS-2B lung epithelial cells, transient knockdown of Gene 33 with siRNA has limited effect on cell cycle progression under the normal culture condition [28]. CRISPR/Cas9-mediated knockout of *ERRFI1* even slightly slows down cell proliferation, although chronic exposure to Cr(VI) eliminated this effect [81]. In addition, no significant change in cell proliferation was observed in rectal cancer cells after Gene 33 depletion [82]. Moreover, Gene 33 expression does not affect proliferation of melanoma cells containing mutant NRAS [83]. Similarly, ectopic expression of Gene 33 does not affect cell cycle distribution and the levels of cyclin D1, p21, and p27 in MCF-7 breast cancer cells [84]. In contrast, Gene 33 knockdown significantly inhibits proliferation PC9 and PC9GR lung adenocarcinoma cells [85]. Moreover, a recent study shows that Gene 33 knockdown promotes the proliferation of MCF10A breast epithelial cells and MDA-MB-468 breast cancer cells while inhibiting that of MDA-MB-231 breast cancer cells [86]. Thus, the effect of Gene 33 on cell proliferation appears to be highly cell type dependent.

The mechanism underlying the differential effect of Gene 33 in cell proliferation is complex. It is possible that this depends on how important EGFR signaling is for the proliferation of a particular cell type. Under the normal culture condition, other growth factors may sufficiently complement the role of EGF in inducing mitotic signaling in some cell types, thereby diminishing the role of Gene 33. This point is supported by a recent study showing that the expression level of EGFR can determine the effect of Gene 33 on cell proliferation [37]. In cancer cells with high levels of Gene 33 expression, Gene 33 knockdown promotes cell proliferation by increasing EGFR signaling, whereas in cells with low EGFR expression, Gene 33 depletion inhibits cell proliferation by enhancing AKT dephosphorylation [37]. In contrast, Gene 33 may directly interact with AKT and inhibit its phosphorylation, thereby suppressing the proliferation of endometrial epithelial cells [38]. The multifunctional nature of Gene 33 may also contribute to the context-dependent role of Gene 33 in cell proliferation by regulating multiple pathways.

### 4.2. Gene 33 in Apoptosis and Senescence

Gene 33 has been shown to promote apoptosis in a number cell types, including cardiomyocytes, pancreatic β cells, glioblastoma cells, mammary epithelial cells, BEAS-2B lung epithelial cells, and A549 lung adenocarcinoma cells [17,27,33,55,87]. Gene 33 induces apoptosis through at least two distinct mechanisms: by inhibition of the EGFR pathway and its associated activation of the AKT, and by c-Abl-dependent activation of p73 [17,27,33]. Gene 33 may also have the potential to induce apoptosis by activating NF-κB in a context-dependent fashion [36]. Overexpression of Gene 33 in rat neonatal cardiomyocytes triggers strong apoptosis by inhibiting ErbB2-induced ERK and AKT pathways [17]. Knockdown of Gene 33 inhibits apoptosis induced by acute hypoxia and hypoxia-reoxygenation [17]. In mammary epithelial cells, Gene 33 mediates apoptosis induced by deprivation of ErbB ligands via the c-Abl-p73 pathway [33]. Specifically, ligand deprivation leads to dissociation of Gene 33 from the ErbB receptor and subsequent binding to c-Abl, thereby increasing its tyrosine kinase activity and promoting its nuclear translocation [33]. Nuclear c-Abl promotes apoptosis by phosphorylating p73, a homologue of p53 [33,88,89,90]. This mechanism may also contribute to apoptosis induced by Gene 33 in lung epithelial cells and lung cancer cells [27]. The ability for Gene 33 to induce apoptosis requires its EBD domain, as this domain is required for its binding and activation of c-Abl [27,33]. Gene 33 also induces apoptosis of uterine epithelial cells by downregulation of baculoviral inhibitors of apoptosis repeat-containing 1 (BIRC1), an estrogen-induced apoptosis inhibitor, and by inhibition of ERK2 phosphorylation [91]. Progesterone treatment leads to increased Gene 33 expression at both mRNA and protein levels, which corresponds to reduced viability of progesterone receptor-positive human endometrial carcinoma cells [76]. Consistently, overexpression of Gene 33 induces apoptosis whereas silencing of Gene 33 prevents the effect of progesterone on the viability of these cells [76,77]. Another study shows that Gene 33 promotes autophagy of hepatocellular carcinoma cells by activating JNK [92].

Antiapoptotic or neutral roles of Gene 33 have also been reported. Gene 33 inhibits apoptosis of MCF-7 cells [84]. In human lung microvascular endothelial (HMVEC-L) cells, knockdown of Gene 33 promotes apoptosis [93]. Gene 33 overexpression increases the viability of PC9 lung adenocarcinoma cells [85]. A recent study showed that Gene 33 activates AKT by preventing its dephosphorylation by PHLPP, which conceivably contributes to the antiapoptotic function of Gene 33 [37]. On the other hand, Gene 33 expression has no effect on apoptosis of melanoma cells containing oncogenic N-Ras mutant [83]. Thus, as in cell proliferation, the role of Gene 33 in apoptosis is also context dependent. This feature is consistent with the notion that Gene 33 is unlikely a core component of the cellular apoptotic pathway and that Gene 33 may influence multiple signaling pathways. The complex interaction may help tip the balance between cell death and survival depending on factors, such as the cell type and expression levels of Gene 33 or other relevant cellular components, i.e., EGFR. For instance, Gene 33 was shown to compete with IκBα for binding to NF-κB through its CRIB domain, thereby activating NF-κB by preventing its degradation [36,94]. NF-κB is well-established to have, depending on the stimuli, an anti-apoptotic function [95,96]. Moreover, a recent study shows that Gene 33 knockdown increases survival and viability of MCF10A breast epithelial cells and MDA-MB-468 breast cancer cells while it inhibits these activities in MDA-MB-231 breast cancer cells [86].

The involvement of Gene 33 in cell senescence has been demonstrated in two reports [97,98]. One study showed that Gene 33 can be upregulated during senescence in human embryonic lung diploid fibroblasts’ 2BS cells in an FOXO3A-dependent fashion [98]. Overexpression of Gene 33 triggered premature senescence manifested as reduced DNA synthesis and appearance of markers for senescence, whereas depletion of Gene 33 delayed the initiation of Ras-induced senescence by inhibiting the EGFR signaling [98]. Another study showed that Gene 33 overexpression led to premature senescence in WI-38 human diploid lung fibroblasts, which was accompanied by reduced phosphorylation of Rb at Ser 249/Thr252 [97]. The study further showed that Gene 33 inhibits B-Raf-V600E-induced senescence by limiting its phosphorylation of Rb at Ser249/Thr252 [97].

### 4.3. Gene 33 in Cell Migration and Invasion

The ability of Gene 33 to interact with the small G-protein CDC42 suggests that Gene 33 may regulate actin organization and cell migration. Indeed, Gene 33 negatively regulates Met-mediated cell migration and neurite outgrowth in a CRIB-domain and CDC42-dependent manner [35,62]. It may also inhibit EGF-induced filopodia formation and cell migration in tumor cells via a similar mechanism [99]. Furthermore, Gene 33 expression exhibits an inverse relationship with the expression of metalloproteases MMP-2 and MMP-9 in lung cancer cells, suggesting that it may inhibit EGF-induced invasion [100]. Depletion of Gene 33 promotes cell migration and invasion of melanoma cells containing N-Ras mutation in response to EGF stimulation [83]. Increased cell migration has also been reported in lung epithelial cells after CRISPR/Cas9-mediated knockout of *ERRFI1* [27]. Overexpression of Gene 33 inhibits invasion of endometrial and thyroid cancer cells [62,78]. In contrast, Gene 33 overexpression increases migration of PC9 lung adenocarcinoma cells [85], suggesting the role of Gene 33 in cell migration may also be context dependent.

### 4.4. Nuclear Functions of Gene 33

Gene 33 has long been viewed as a cytoplasmic protein, which carries out its cellular function by interacting with other proteins in the cytoplasm, although hints of its nuclear localization were reported in earlier studies [62,100]. Recent work has revealed nuclear functions of Gene 33 [27]. Nuclear Gene 33, albeit a small fraction, is associated with the chromatin and promotes chromatin association of c-Abl [27]. Ectopic Gene 33 expression triggers the DNA damage response (DDR) in an ATM-dependent fashion [27,28]. Gene 33 binds to histone H2AX and promotes its interaction with ATM [27]. Given the previous finding that c-Abl enhances DDR by phosphorylating ATM [101,102,103,104], Gene 33 may facilitate DDR via a c-Abl-dependent mechanism. Gene 33 may also regulate DDR through its binding to chromatin and chromatin-regulating proteins, as well as by regulating the machinery for DNA repair and synthesis. Paradoxically, depletion of Gene 33 increases DDR induced by genotoxic agents, such as (Cr(VI)) and cisplatin [27,28]. The possible explanation of these observations is that binding of ectopically expressed Gene 33 triggers chromatin disturbance that mimics DDR while depletion of Gene 33 increases DNA damage induced by genotoxic agents as a result of reduced DNA repair [27]. In addition, nuclear localization of Gene 33 provides a spatial explanation for its regulation of the c-Abl/p73 pathway, as this mechanism primarily occurs in the nucleus [27,33]. 

Nuclear localization of Gene 33 is apparently regulated by its 14-3-3BD, as the point mutation that substitutes the key serine residue (S251, Figure 2A) within the domain greatly increases its nuclear localization [27]. Phosphorylation of 14-3-3BD is essential for its binding to 14-3-3, which is a common cellular mechanism for cytosolic retention of proteins containing 14-3-3BD [105,106]. This mechanism could regulate translocation of Gene 33 between cytosol and the nucleus. The kinase that phosphorylates the 14-3-3BD of Gene 33 identified to date is CHK1 [39,40]. Given the well-established role of CHK1 cell cycle checkpoint regulation in response to DDR [107,108], this finding agrees with the notion that Gene 33 has DDR-associated nuclear functions [27]. Supporting this, Gene 33 expression appears to be associated with increased radio-resistance in cancer cell lines [82]. The discovery of its nuclear function highlights a new direction in understanding the overall function of Gene 33.

### 4.5. Gene 33 in Development and Homeostasis

Studies on *Errfi1* knockout mice have provided important insights into its role in development. Whole-body *Errfi1* knockout mice, although born normal, develop degenerative joint disease resembling human osteoarthritis as a result of mesenchymal progenitor cell over-proliferation and die within 6 months of age [72,73,74,75]. Cartilage-specific deletion of *Errfi1* in mice confirms the critical role of Gene 33 in regulating the chondrocyte activity and maintaining joint homeostasis [74,109,110]. Interestingly, cartilage-specific overexpression of *Errfi1* also accelerates cartilage degeneration, which leads to osteoarthritis in mice at an older age of 12–18 months [111]. Furthermore, hypoxia-induced Gene 33 expression via HIF-2 promotes the differentiated phenotype of human articular chondrocyte, thereby regulating cartilage homeostasis [112]. Gene 33 may also contribute to the inhibitory effect of prenatal dexamethasone exposure on endochondral ossification in the long bones of rats [113]. These data indicate that optimal expression of Gene 33 is critically important for maintaining joint homeostasis. 

Mice with *Errfi1* deletion exhibit abnormal skin morphogenesis resembling transgenic expression of TGF-α, a ligand of EGFR [20]. These observations are consistent with overactive EGFR signaling in skin keratinocytes [20]. Conversely, forced skin-specific expression of Gene 33 in mice leads to a wave-like phenotype mimicking the skin morphology of mice with hypomorphic or antimorphic EGFR alleles [114]. *Errfi1* knockout mice also exhibit abnormal lung development, which results in partial embryonic lethality at the perinatal stage [75,93]. 

The *Errfi1* mRNA level is strongly associated with the early development of rats. The level of Gene 33 mRNA is low on E19, increases 3-fold between E19 and 21, doubles again at birth, and returns to the base level one day after birth [115]. The sharp but transient increase of *Errfi1* expression immediately after birth is particularly prominent in the liver, kidney, and lung [93,116,117], suggesting that Gene 33 may be important for the postnatal development of these tissues. However, careful examination revealed only abnormal postnatal development of the lung in *Errfi1*-null mice [93]. These data indicate that Gene 33 is important for postnatal development of the lung but not the liver and kidney. Gene 33 seems to also be important for the development of the uterus by mediating the inhibitory effect of progesterone toward estrogen [51]. Thus, progesterone induces expression of Gene 33, which in turn blunts the response of endometrium to estrogen [51]. This mechanism is apparently critical for uterine implantation in mice [118].

In contrast to its benign role in liver development, Gene 33 seems to play a significant role in liver regeneration. *Errfi1* is dramatically induced in the liver immediately after partial hepatectomy in rats and *ERRFI1* is strongly induced in the liver of patients suffering fulminant hepatic failure [119]. In *Errfi1* knockout mice, EGFR expression is elevated in the liver, which correlates with increased liver regeneration after partial hepatectomy [26]. Primary hepatocytes from *Errfi1* knockout mice display more sustained mitogenic signals in the early phase of partial hepatectomy [26]. Moreover, expression of Gene 33 is inversely associated with the survival of patients after hepatectomy [120]. Given the importance of the EGF and c-Met pathways in both processes [121], the differential role of Gene 33 in postnatal liver development and liver regeneration is interesting. One speculation would be that the EGFR and c-Met pathways are relatively irreplaceable for regeneration from acute liver damage whereas other signaling pathways, such as the Wnt pathway, may compensate most of the functions conferred by the EGFR and c-Met pathways [122]. Further studies are clearly needed to answer this question. 

**Figure 4:**
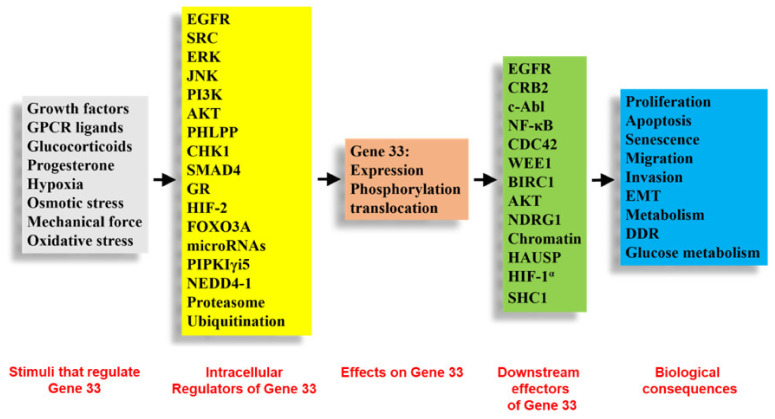
Summary of the known regulations and biological functions of Gene 33. Abbreviations: EMT: epithelial mesenchymal transition, DDR: DNA damage response. Other abbreviations are gene names.

Gene 33 has been shown to participate in cholesterol homeostasis and bile acid synthesis in the liver [123]. Liver-specific conditional knockout of *Errfi1* in mice leads to hepatomegaly and fatty liver, which is accompanied by elevated serum levels of LDL, HDL, and hepatic lipid [123]. Bile acid excretion is also lower in the knockout mice [123]. DNA microarray analysis revealed changes in gene expression that are consistent with the role of Gene 33 in cholesterol and bile acid synthesis as well as lipid metabolism [123]. A meta-analysis of genome-wide association studies revealed that Gene 33 may play a role in hair shape development [124]. A recent study shows that Gene 33 interacts with the adapter protein SHC1, thereby inhibiting its tyrosine phosphorylation at Tyr239/240 and its pro-angiogenic function [43]. Figure 4 summarizes the known regulations and functions of Gene 33. 

## 5. Gene 33 and Human Diseases

### 5.1. Gene 33 in Cancer

The involvement of Gene 33 in the signaling of receptor tyrosine kinases of the EGFR family prompted immediate interest in its potential role in cancer, as these receptors are heavily involved in various types of human cancer [125]. The function of Gene 33 as an inhibitor of these receptors suggests a potential tumor suppressive role of this protein. Other known functions of Gene 33, including the proapoptotic and the nuclear function of Gene 33, are also largely in line with this notion. Indeed, *Errfi1*-null mice develop neoplasms in multiple tissues [20]. In humans, *ERRFI1* is located in chromosome 1p36, a locus long believed to contain multiple putative tumor suppressor genes and frequently altered in many cancers [126]. These data support a general role of Gene 33 in human cancer as a tumor suppressor.

#### 5.1.1. Gene 33 in Lung Cancer

Strong evidence indicates that Gene 33 is a bona fide tumor suppressor in the lung. In humans, deletion of the 1p36 locus of the chromosome, where *ERRFI1* is located, occurs in nearly 50% of human lung cancer [127]. Loss of heterozygosity (LOH) of this region occurs frequently in human lung cancer and is associated with smoking patients, squamous cell carcinoma patients, and late-stage patients [128]. The corresponding locus in mice is also frequently subjected to LOH in spontaneous and carcinogen-induced lung adenoma [129,130,131]. *Errfi1* knockout causes spontaneous lung tumorigenesis in mice [20]. Although human lung cancer cell lines express various levels of Gene 33, some cell lines, e.g., NCI-H226 and NCI-H322, lack this protein [132]. Mechanistically, NCI-H226 expresses little *ERRFI1* mRNA due to dysregulation of mRNA transcription while NCI-H322 cells express a high level of *ERRFI1* mRNA containing a nonsense mutation at codon 83, which renders it undetectable by the Gene 33 antibody targeting the C-terminus of the protein due to truncation [132]. Some human lung cancer cell lines, e.g., NCI-H23 cells, express a very high level of Gene 33 due to an overactive ERK pathway as a result of activating mutant of *RAS* [132]. Expression of Gene 33 in lung cancer cells can also be affected by the level of EGFR [132]. Interestingly, stable ectopic expression of the wild-type EGFR increases the *ERRFI1* mRNA level but not the protein level whereas ectopic expression of the L858R mutant EGFR, which is a common EGFR mutant in lung cancer, elevates both the mRNA and protein levels of Gene 33 in H1299 lung cancer cells [133]. These results highlight the disconnection of the mRNA and protein levels of Gene 33 and the importance of keeping the Gene 33 protein level in check to prevent overactivation of EGFR in lung cancer cells.

Genetic analyses revealed polymorphisms and missense mutations of the *ERRFI1* gene in some human lung cancer cell lines and primary lung cancer cells [132]. In human lung adenocarcinoma patients, potential oncogenic nonsense mutations at serine 177 and 441of *ERRFI1* exist, albeit at relatively low frequencies of ~0.15% (the TCGA database). Other potential oncogenic changes of *ERRFI1* in human lung cancer patients, although at even lower frequencies, include frameshift insertions at serine 372 and threonine 187, and the fusion with *DNAH2* (the TCGA database). A germline mutation (A373V) has also been detected in a lung cancer patient [132].

Consistent with the genetic changes, downregulation of Gene 33 protein expression is common in human NSCLC. More than 50% of NSCLC exhibits lower Gene 33 expression and the downregulation is associated with poor differentiation of the cancer [100]. Interestingly, nuclear but not cytoplasmic immunohistochemical staining of Gene 33 is correlated with the histology, differentiation, and status of these tumor specimens [100]. Gene 33 expression has an inverse relationship with tyrosine phosphorylation of EGFR and the expression of metalloproteases MMP-2 and MMP-9 in lung cancer cells, suggesting a role of Gene 33 in lung cancer cell migration, invasion, and metastasis [100].

Loss of Gene 33, both homozygous deletion and haploinsufficiency, accelerates the initiation and progression of lung adenocarcinoma driven by transgenic expression of mutant EGFRs (L858R or del-746-750) in mice, likely a result of increased MAPK activity due to reduced inhibition of EGFR [34]. Of note, expression of the EGFR mutants is associated with lower levels of Gene 33 protein but higher levels of phosphorylated EGFR, which lead to a higher ratio of phosphorylated/total EGFR levels [34]. Thus, the higher ratio of phosphorylated EGFR/total EGFR may be responsible for the increased tumorigenesis [34]. In addition, mutant EGFR is resistant to Gene 33 mediated- and lysosome-dependent degradation [34]. Gene 33 deletion also promotes lung tumorigenesis induced by oncogenic K-Ras in mice: *Errfi1* knockout mice in the *Kras*^G12D^ strain background have increased levels of lung tumorigenesis compared to *Kras*^G12D^ mice [134]. The increased lung tumorigenesis is accompanied by elevated levels of inflammatory response and phosphorylated ErbB4 as well as a reduced level of apoptosis [134]. Gene 33 has also been shown to play an important role in lung tumor progression in *Pten* and *Smad4* double knockout mice by inhibiting ErbB2, as Gene 33 appears to be a downstream target of SMAD4 [135]. Gene 33 has also been shown to inhibit lung epithelial cell transformation induced by genotoxic carcinogens, such as hexavalent chromium (Cr(VI)), at least in part by regulating DDR [28,81]. CRISPR/Cas9-mediated deletion of Gene 33 combined with chronic treatment with low-concentration Cr(VI) leads to change in the gene expression pattern of lung epithelial cells consistent with the increased transformation [81].

#### 5.1.2. Gene 33 in Endometrial Cancer

A strong connection between Gene 33 and endometrial cancer has been established by a series of studies, particularly from Dr. Jae-Wook Jeong’s laboratory. Cre/flox-mediated knockout of *Errfi1* leads uterine hyperplasia [136]. Ovariectomized *Errif1* knockout mice treated with estrogen develop invasive endometrial adenocarcinoma after 3 months while those treated with both progesterone and estrogen only develop endometrial hyperplasia [136]. Progesterone is known to counter the effect of estrogen in endometrial dysfunction and progestin, a synthetic mimic of progesterone, has been used as therapy for endometrial hyperplasia and endometrial carcinoma [137,138]. Since Gene 33 expression in the endometrial tissue is highest in the secretory phase of the menstrual cycle, which is correlated with high progesterone levels [76,136], these data support the notion that Gene 33 may contribute to the inhibitory effect of progesterone on estrogen-induced endometrial carcinogenesis in humans. Supporting this, Gene 33 expression is significantly lower in human endometrioid carcinoma compared to endometrial tissues in the secretory phase of the menstrual cycle [77,136]. In addition, progesterone promotes Gene 33 expression at both the mRNA and protein levels, which corresponds to reduced viability of progesterone receptor-positive human endometrial carcinoma cells [76]. Silencing of Gene 33 prevents the effect of progesterone on cell viability, indicating that Gene 33 mediates progesterone-mediated growth suppression in human endometrial cancer cells [76]. Conversely, overexpression of Gene 33 induces apoptosis and inhibits proliferation and invasion of endometrial cancer cells [77]. Moreover, Gene 33 overexpression enhances the inhibition of estrogen-induced effects by progesterone in human endometrial carcinoma cells [77].

Further supporting a role of Gene 33 in endometrial cancer, tissue-specific knockout of *Errfi1* from mouse uterine epithelium using the *Wnt7a*-Cre/*Errfi1*^flox/flox^ approach promotes endometrial hyperplasia and estrogen-dependent endometrial cancer, indicating that epithelial Gene 33 expression is critical for the development of endometrial malignancy and that progesterone in the stroma may inhibit the neoplastic transformation of uterine epithelial cells [139]. Furthermore, uterus-specific double knockout of *Pten* and *Errfi1* produced a synergistic effect on endometrial tumorigenesis [91], suggesting a tumor-suppressive mechanism of Gene 33 that is independent of the PTEN-mediated pathway in endometrial tumors. Consistently, transgenic overexpression of Gene 33 inhibits uterine tumorigenesis caused by *Pten* deletion in mice [140].

#### 5.1.3. Gene 33 in Glioma

As in lung cancer, chromosome 1p36, where *ERRFI1* is located, is among the genetic loci that are frequently deleted in glioblastoma [25,141]. More detailed analysis revealed that the most commonly deleted region within chromosome1p36.23 contains only *ERRFI1* [141]. Although homologous deletion of chromosome 1p36 occurs in only 15 out of 430 glioblastoma samples, up to 50% of primary glioblastoma samples and cell lines have downregulated expression of Gene 33 [25,141]. Ectopic expression of Gene 33 in the H423 glioblastoma cell line with homozygous deletion of *ERRFI1* suppresses cell migration [141]. In addition, miR-148 regulates EGFR activity by targeting Gene 33 in glioblastoma [55]. The deletion mutant of EGFR with constitutively active tyrosine kinase activity (EGFRvIII) is common in glioblastoma and contributes significantly to cancer cell growth and survival [142]. This mutant is a result of loss of exons 2–7 of the *EGFR* gene, which leads to an in-frame deletion [142]. This EGFR mutant appears to be much more able to phosphorylate Gene 33 at tyrosine 394 [143], a residue associated with reduced inhibition of EGFR when phosphorylated [34,68]. Moreover, 47.4% of glioblastoma patient samples show coexistence of EGFRvIII and loss of Gene 33 [25]. These data indicate that *ERRFI1* is a significant tumor suppressor gene in glioblastoma. 

#### 5.1.4. Gene 33 in Papillary Thyroid Cancer

Reduced Gene 33 expression has been reported in 79% of specimens of papillary thyroid cancer patients, likely a result of methylation of the *ERRFI1* promotor [62]. Low Gene 33 expression is correlated with low activity of NF-kB but high levels of EGFR and ERK phosphorylation in these specimens [62]. The study on thyroid-specific *Errfi1* knockout mice confirmed this signaling pattern [94]. Gene 33 expression also inversely correlates with the size of the tumors [62]. Higher Gene 33 expression is associated with longer survival and disease-free survival of papillary thyroid cancer patients, as well as an independent predictor of better disease-free survival in papillary thyroid cancer containing the valine to glutamate mutation at amino acid 600 of BRAF (BRAFV600E) [144]. In contrast, decreased expression of *ERRFI1* is associated with a more aggressive phenotype of papillary thyroid cancer with *BRAF* mutation [71]. In thyroid cancer cell lines, Gene 33 knockdown enhances the proliferative signaling, reduces apoptosis, increases activities of metalloproteinase-2 and-9, and promotes cell invasion [62]. These findings are in line with the general functional profile of Gene 33 and support a potential tumor suppressive role of Gene 33 in papillary thyroid cancer.

#### 5.1.5. Gene 33 in Breast Cancer

Although LOH of chromosome 1p occurs in 40–60% of breast tumors [145,146], *ERRFI1* is not among the genes that are commonly deleted [24]. In addition, no mutation was detected in the coding sequence of *ERRFI1* in these tumors [24]. However, underexpression of Gene 33 appears to be common in invasive breast carcinoma, particularly triple-negative breast carcinoma [147]. Examination of a panel of breast cancer cell lines revealed a generally positive correlation between the level of Gene 33 and that of ErbB2, except two with very high ErbB2 levels but very low levels of Gene 33 [24]. Ectopic expression of Gene 33 in these two cell lines inhibits their proliferation [24]. These data suggest that loss of Gene 33 expression may contribute to progression of some breast cancer by elevating the activity of ErbB2. Consistently, microarray analysis revealed a positive relationship between *ERRFI1* expression and breast cancer patient survival [148]. Interestingly, while Gene 33 acts as a tumor suppressor in in situ breast tumor cells, it is required for prevention of apoptosis and for metastasis of MDA-MB-231 breast cancer cells by inhibiting EGF-dependent inhibition of metastasis [149]. Supporting the differential role of Gene 33 in breast cancer cell lines, a recent study showed that Gene 33 knockdown tends to promote survival, proliferation, and viability of MCF10A breast epithelial cells and the triple-negative breast cancer (TNBC) cells MDA-MB-468 while it inhibits these activities in the more aggressive TNBC MDA-MB-231 cells [86]. Another study showed that breast cancer cell lines express various levels of Gene 33 and that ectopic expression of Gene 33 inhibits apoptosis of MCF-7 cells without affecting cell proliferation [84]. A recent study indicates that Gene 33 may have an important role in the metabolism of TNBC cells [150]. The study showed that Gene 33 is upregulated in TNBC and positively correlated with poor clinical outcomes of the patients. Using metabolism and functional arrays, the study found that Gene 33 drives the reprogramming of glucose metabolism toward glycolysis. Mechanistically, Gene 33 recruits the HAUSP deubiquitinase to stabilize HIF-1α, thereby upregulating GLUT1 and other HIF-1α-regulated glycolytic genes [150]. These data suggest complex and differential roles of Gene 33 in different types and stages of breast cancer progression.

#### 5.1.6. Gene 33 in Skins Cancer

Several lines of evidence support a role of Gene 33 in skin cancer. *Errfi1*-null mice exhibit skin hyperplasia and increased susceptibility to carcinogen-induced skin papilloma and melanoma [20]. Gene 33 inhibits migration and invasion of mutant N-Ras-driven melanoma cells after MEK inhibition with trametinib without affecting the proliferation, anchorage-independent growth, and survival of these cells [83]. These data are consistent with the role of Gene 33 as a tumor suppressor in melanoma. Reduced Gene 33 expression and the resulting increase in the EGFR-Ras-ERK pathway may contribute to keratinocytes’ hyperproliferation and tumorigenesis caused by lowered expression of CREB-binding protein (CBP) and p300, two closely related lysine acetyltransferases [151]. In contrast, tissue microarray analysis revealed significantly higher expression of Gene 33, which is associated with earlier death of patients with metastatic melanoma [152]. This finding suggests the Gene 33 may promote metastatic melanoma, as shown in metastatic breast cells [149].

#### 5.1.7. Gene 33 in Other Cancer Types

Gene 33 has also been implicated in other cancers. A tumor suppressive role of Gene 33 has been reported in the liver. Gene 33 inhibits EGF-induced cell migration of human liver cancer cell lines [26]. Reduced expression of Gene 33 was observed in human hepatocellular carcinoma, which correlated with increased EGFR expression, activation of the ERK-cyclin D pathway, and expression of miR-589-5p [26,60,153]. In addition, inhibition of Gene 33 expression by miR-214 contributes to liver fibrosis and tumorigenesis in PDGF transgenic mice [57].

Gene 33 is a prognostic marker for colorectal cancer patients with liver metastases and is negatively associated with patient survival [120]. Again, these data support a positive role of Gene 33 in cancer metastasis, as in breast and melanoma [149,152]. On the other hand, Gene 33 seems to mediate the anti-metastatic function of N-Myc downstream-regulated gene 1 (NDRG1) in pancreatic cancer, as increased expression of NDRG1 posttranscriptionally stabilizes Gene 33, thereby inhibiting EGFR-dependent metastasis by promoting lysosomal degradation of EGFR [67]. This discrepancy could be a result of the differential requirement for EGFR signaling at a different stage of the metastasis and/or different cancer types. Nonsense mutation at glutamic acid 384, within the segment 2 of the EGFR and c-Abl binding motif of *ERRFI1* [22,33], exists in sporadic intrahepatic cholangiocarcinoma and is associated with enhanced cancer regression upon EGFR inhibition [154]. Moreover, a recent study shows that the long noncoding RNA *ANRIL* suppresses the expression of Gene 33, thereby promoting the progression of cholangiocarcinoma [155]. 

#### 5.1.8. Gene 33 in Acquired Resistance to Cancer Therapy

Mounting evidence indicates that Gene 33 plays an important role in acquired resistance to targeted therapy using EGFR tyrosine kinase inhibitors (TKIs). Loss of Gene 33 expression, which is associated with higher ErbB2 activity, confers resistance to the EEBB2 neutralizing antibody Herceptin in breast cancer [24]. Consistently, overexpression of Gene 33 is associated with higher sensitivity of NSCLC cells to the EGFR inhibitor gefitinib [156]. However, a high expression ratio between Gene 33 and EGFR is positively associated with resistance to the EGFR inhibitor erlotinib by a panel of cancer cells, including human NSCLC cells, bladder cancer cells, and head and neck squamous cell carcinoma cells [157]. Similar trends were also observed in human pancreatic cancer patient-derived xenograft (PDX) models and NSCLC specimens in resistance to erlotinib or gefitinib [157]. Thus, low activity of EGFR, as a result of a high Gene 33/EGFR ratio, rather than the absolute level of Gene 33 or EGFR predicts high resistance to EGFR inhibition [157]. Of note, induction of Gene 33 by the PI3K-AKT pathway leads to a higher Gene 33/EGFR ratio in the resistant cells [157]. Gene 33 knockdown renders EGFR-TKI-resistant PC9/GR lung adenocarcinoma cells sensitive to EGFR-TKI inhibition [85].

Using a cancer tissue-originated spheroid (CTOS) system, Endo et al. demonstrated that primary human lung cancer cells with activating mutation of EGFR (L858R) enter a dormant state under hypoxia [158]. These cells exhibit constitutive phosphorylation of EGFR, increased expression of Gene 33, and resistance to EGFR inhibition therapy [158]. The increased Gene 33 expression promotes the resistance to EGFR inhibition by preventing heterodimer formation of the ErbB family receptor tyrosine kinases [158]. These findings highlight an interesting and important way for Gene 33 to regulate the resistance to EGFR inhibition therapy.

Gene 33 may also mediate resistance to MET-TKI therapy. In MET overactive (as a result of MET amplification and constitutive activation) gastric carcinoma cells and lung squamous cells treated to be resistant to MET-TKI, miR-205 is epigenetically induced [159]. As a target of miR-205, Gene 33 expression is suppressed, which eases the inhibition of EGFR and leads to EGFR activation. The activation of EGFR results in resistance to MET-TKI [159]. In epithelial mesenchymal transition (EMT) mediated by TGF-β, the expression of the miR-200 family microRNAs and miR-205 are suppressed to facilitate the EMT process [160]. As a target of these microRNAs, Gene 33 expression is increased, which in turn inhibits EGFR to promote EMT [54]. This process happens along with the TGF-β-induced EMT and helps switch cells to an EGFR-independent state that is resistant to EGFR-TKI [54]. This mechanism appears to apply to a panel of lung cancer, bladder cancer, and head and neck cancer cell lines [54]. Conversely, ectopic expression of miR-200 reverses the resistance of bladder cancer cells to EGFR inhibition therapy by regulating EMT [161]. Gene 33 may also contribute to resistance of lung cancer stem cells to the EGFR TKI by suppressing their transition from self-renewal to malignant differentiation, a process dependent on EGFR signaling [162].

Gene 33 has also been shown to participate in the resistance to inhibitors of the members of the MAP kinase pathway. Resistance to MEK inhibition in gastric and colorectal cancer cells with mutated *KRAS* occurs as a result of downregulation of Gene 33 by MEK inhibition, which increases EGFR signaling to AKT by reducing the inhibition of EGFR by Gene 33 [163]. Similarly, mutated B-Raf induces Gene 33 expression, which in turn inhibits EGFR in papillary thyroid cancer [71]. Inhibition of B-Raf by vemurafenib leads to reduced expression of Gene 33 and increased EGFR activity [71]. This mechanism may contribute significantly to the resistance of these cancers to the K-Ras or B-Raf inhibition therapy [71,163]. Finally, Gene 33 is induced in radio-resistant rectal cancer cells, which may contribute to the resistance to radiation therapy of these cells [82].

### 5.2. Gene 33 in Diabetes

*Errfi1* was shown initially by the laboratory of Joseph Messina to be an insulin-inducible gene in H4 hepatocarcinoma cells soon after its discovery [164]. Follow-up studies confirmed this observation [2,8,50,52,164]. As in other settings, the induction of Gene 33 by insulin is MEK-ERK dependent [52]. These findings suggest a potential role of Gene 33 in the development of diabetes. Supporting this notion, Gene 33 expression is strongly induced in a murine model for diabetic nephropathy and was proposed to mediate diabetic renal hypertrophy [4]. Furthermore, a study based on a Korean population revealed a single nucleotide polymorphism (SNP) in the third intron of the *ERRFI1* gene (+808(T/G)) that is negatively associated with diabetic nephropathy [165]. This SNP is believed to negatively regulate the expression of Gene 33 [165].

*Errfi1* haploinsufficiency protects mice from streptozotocin-induced diabetes [166]. This prodiabetic function of Gene 33 appears to be a result of the ability of Gene 33 to promote DNA damage-induced apoptotic death of pancreatic β cells in response to proinflammatory cytokines and ER stress [87,166]. Gene 33 may also mediate glucocorticoid-induced insulin resistance and diabetes by suppressing β cell proliferation via inhibition of EGFR signaling [79]. In contrast, mice with liver-specific knockout of *Errfi1* exhibit hyperglycemia as a result of hepatic insulin resistance [167]. Deletion of *Errfi1* increases EGFR signaling, mTOR activity, JNK activity, and IRS-1 phosphorylation at serine 307 [167]. On the other hand, hepatic expression of glucokinase, glucose-6-phosphatase, and phosphoenolpyruvate carboxykinase 1 is reduced after *Errfi1* ablation [167]. Interestingly, another study showed that liver-specific knockout of *Errfi1* in mice leads to fatty liver, fasting hyperglycemia, and hypercholesterolemia but lower body weight and higher insulin sensitivity [168]. The hypercholesterolemia in the knockout mice is apparently a result of increased EGFR activation, as inhibition of EGFR with TKI reduces hypercholesterolemia [169]. These findings highlight a rather complex role of Gene 33 in diabetes and metabolic syndrome, and possible differential roles in conditions mimicking type 1 and type 2 diabetes.

### 5.3. Gene 33 in Cardiovascular Diseases

ErbB2 signaling is well established to promote cardiomyocyte survival while inhibition of it leads to cardiomyocyte apoptosis and dilated heart failure [170,171,172,173,174]. Gene 33 overexpression using the adenoviral vector leads to strong apoptosis of rat neonatal myocytes as a result of inhibition of the antiapoptotic AKT and ERK activity downstream of ErbB family tyrosine kinases [17]. Gene 33 is induced in rat neonatal myocytes by hypoxia and hypoxia/reoxygenation and in turn promotes apoptosis [17]. Moreover, Gene 33 is strikingly induced in the murine cardiac tissue suffering experimentally induced myocardial infarction or ischemia and ischemia/reperfusion [17]. These findings point to a significant role of Gene 33 in the loss of cardiomyocytes during ischemia induced by myocardial infarction by promoting cardiomyocyte apoptosis.

On the other hand, constitutive cardiac-specific ectopic expression of Gene 33 in mouse heart attenuates both hypertrophic and inflammatory responses and helps preserve cardiac functions [175]. This is accomplished through inhibition of the angiotensin II and isoproterenol-stimulated vasoactive signaling pathways, which are known to transactivate EGFR [70,175]. Unlike in the case of ischemic stress, transgenic expression of Gene 33 does not cause significant cardiomyocyte apoptosis in mice, highlighting the differential role of Gene 33 in acute and chronic cardiac conditions [17,175].

Using smooth muscle-specific *Errfi1* knockout mice and the cellular model, Lee et al. showed that Gene 33 inhibits EGF-dependent smooth muscle proliferation and migration that lead to neointimal hyperplasia, thereby potentially inhibiting atherosclerosis [176]. 

### 5.4. Gene 33 in Other Diseases 

Gene 33 has been documented to promote intervertebral disk degeneration, in which miR-2355 is upregulated and suppresses the expression of Gene 33 [56]. Gene 33 in turn inhibits EGFR-mediated proliferation of intervertebral disc cells, particularly nucleus pulposus cells [56]. Microarray analyses detected upregulation of *ERRFI1* in the central nervous system after acute infection by pseudorabies virus, suggesting a potential involvement of Gene 33 in the response of the central nervous system to viral infection [177]. Downregulation of *Errfi1* was observed in the liver of mice subjected to disrupted growth hormone and insulin-like growth factor-1 signaling as well as caloric restriction, suggesting a potential involvement of Gene 33 in dwarfism and longevity [178]. Interestingly, a reduced plasma level of Gene 33 has been shown to be associated with children with autism [179].

A recent integrated analysis of public genomic data revealed a potential association between *ERRFI1* with the risk of psoriasis, a chronic inflammatory skin condition [180]. An enhancer variant in the chromosome 1p36.23 region and within the enhancer of *ERRFI1* is associated with increased susceptibility for psoriasis. The variant may render the *ERRFI1* enhancer less able to interact with the AP-1 complex at the *ERRFI1* promoter, thereby destabilizing the expression of the gene [180]. Interestingly, psoriasis has been shown to be associated with nonalcoholic fatty liver disease, type 2 diabetes, and metabolic syndrome [181]. These findings provide further support to the involvement of Gene 33 in diabetes and metabolic syndrome. Table 1 summarizes the known association of Gene 33 with human diseases.

## 6. Conclusions and Perspectives

Our understanding of Gene 33 has come a long way since its discovery more than 30 years ago. The nature of this protein as an adapter/scaffold and the relative late appearance in the evolution suggest that its main functions are likely to coordinate multiple cellular pathways in response to the increased biological complexity of higher metazoans. The functions of Gene 33 discovered to date appear to support this notion. It would not be surprising that more functions of Gene 33 will be revealed along this line of reasoning. Recent evidence on its nuclear and chromatin-associated functions in DDR and DNA repair points to a new direction in searching for additional functions of this protein. Although Gene 33 may not be one of the core regulators of these biological processes, its importance in pathophysiological processes should not be overlooked. In fact, many biological processes, chronic pathological conditions in particular, are strongly associated with seemingly weak biological alterations rather than overt biological defects with severe consequences, e.g., early mortality. Therefore, the study of proteins, such as Gene 33, is important for better understanding of these chronic conditions, which could lead to novel therapeutic strategies. For instance, the nuclear function of Gene 33 on DDR may contribute to carcinogenesis induced by genotoxic agents with environmental or occupational relevance, such as hexavalent chromium, by affecting Cr(VI)-induced genomic instability. Gene 33 dysregulation may also facilitate spontaneous gene mutations that lead to genomic instability and carcinogenesis. In diabetes, abnormal function of nuclear Gene 33 could contribute to chronic loss of β cells through dysregulation of cell cycle checkpoint control at the G2/M phase. A similar mechanism could be involved in psoriasis, where abnormal proliferation of skin cells contributes to the symptom of the disease. The nuclear function of Gene 33 adds additional complexity to the already complex functional profile of Gene 33. Further investigation into the role of nuclear Gene 33 in chronic human diseases is clearly needed and potentially fruitful.

## Figures and Tables

**Table 1:** Gene 33 and human diseases.

**Function in Cancer**	**Cancer Type**	**Reference**
Suppressor	Lung cancer	[20,28,34,81,100,132,133,134,135]
	Endometrial cancer	[76,77,91,136,139]
	Glioma	[25,55,141,142]
	Thyroid cancer	[62,71,94,144]
	Liver cancer	[26,57]
Suppressor or promotor	Breast cancer	[24,84,148,149]
	Skin cancer	[20,83,152]
Marker for liver metastasis	Colorectal cancer	[120]
**Function in other diseases**	**Disease**	**Reference**
Suppressor	Cholangiocarcinoma	[154]
promotor	Intervertebral disk degeneration	[56]
Suppressor or promotor	Diabetes	[4,79,87,165,166,167,168]
Suppressor or promoter	Cardiovascular diseases	[17,175,176]
Positive correlation	Dwarfism and longevity	[178]
Negative correlation	Psoriasis	[180]
Negative correlation	Autism	[179]

## Abbreviations

14-3-3-BD	14-3-3– binding domain
AH	Ack homology
CRE	cAMP response element
CRIB	Cdc42/Rac-interactive binding
DEAD	Asp-Glu-Ala-Asp box
DDR	DNA damage response
EBD/EBR	ErbB binding domain/region
EMT	epithelial mesenchymal transition
GR	glucocorticoid receptor
GRE	glucocorticoid response element
HRE	HIF-response element
LOH	loss of heterozygosity
LPA	lysophosphatidic acid
Mig6	mitogen-inducible gene 6
NLS	nuclear localization signal
PDGF	platelet-derived growth factor
PDZ-BD	PDZ binding domain
PEST	Pro, Glu, Ser, Thr-rich sequence
RALT	receptor-associated late transducer
RED	RALT endocytic domain
RTK	receptor tyrosine kinase
SH2	Src homology 2
SH3-BD	Src homology 3 binding domain
SNP	single nucleotide polymorphism
SRE	serum response element
TCGA	the cancer genome atlas
TKI	tyrosine kinase inhibitor
TNBC	triple negative breast cancer
TRE	TPA response element
BRafV600E	valine to glutamate mutation at amino acid 600 of BRAF
